# Compassion‐based interventions in Asian communities: A meta‐analysis of randomised controlled trials

**DOI:** 10.1111/papt.12431

**Published:** 2022-10-31

**Authors:** Lasara Kariyawasam, Margarita Ononaiye, Chris Irons, Sarah E. Kirby

**Affiliations:** ^1^ Department of Psychology University of Southampton Southampton UK; ^2^ Balanced Mind London UK

**Keywords:** Asian, compassion, cross‐cultural, efficacy, intervention

## Abstract

**Purpose:**

Practising compassion increases well‐being and reduces depression, anxiety, and psychological distress among clinical and non‐clinical populations. There is a rapid increase in compassion‐based interventions within the past two decades. However, the reviews are limited to predominantly Western cultures. Therefore, this meta‐analysis aimed to evaluate the literature attempting to promote and increase compassion in Asian communities.

**Method:**

Eight randomised controlled trials (RCTs) conducted between 2016 to 2021 were included in the meta‐analysis with data from 1012 participants across Thailand, Japan, China and Hong Kong. Effect sizes were calculated to test the efficacy of the compassion‐based interventions on the self‐compassion outcome. Intervention efficacy was tested by comparing the intervention groups against control groups (wait‐list control and active control groups) at pre‐ and post‐interventions.

**Results:**

Significant between‐group differences in change scores were found on self‐report measures of self‐compassion with large effect sizes in interventions with wait‐list control groups (*d* = .86) and small effect sizes in interventions with active‐control groups (*d* = .19).

**Conclusions:**

Although compassion‐based interventions are heterogeneous in nature and limited in scope, there is promising evidence of improving self‐compassion in Asian communities. This supports for the cross‐cultural applicability of compassion‐based interventions. However, research within the Asian context is limited and at an infancy stage, signifying the importance of conducting further compassion‐based interventions in clinical and non‐clinical groups living in the Asian communities.

**Practitioner Points**

- There is an increase interest in compassion‐based interventions in Western countries
- Most compassion‐based interventions and Western concepts of compassion are influenced by Eastern philosophies and Buddhist psychology
- Many Asian cultures embrace and practice Buddhism
- However, there is an evident dearth of compassion related studies and compassion‐based interventions in the Asian context
- It is important to conduct compassion‐related studies in Asian communities, considering that compassion has been found to be at least partially determined by culture.

## INTRODUCTION

The concept of compassion has been widely discussed in Buddhism and other practiced religions (Germer & Siegel, 2012; Strauss et al., 2016). Compassion is commonly understood as an openness to consciously turn towards suffering, rather than away from it (Gilbert, 2014a). Whilst compassion‐based meditations have formed a central part of some spiritual traditions (e.g. Buddhism: Lama & Thupten, 1995), they have also been incorporated into treatment approaches in psychotherapy (Gilbert, 2013; Neff, 2003a). Practising compassion has shown increased improvements in psychological and physiological well‐being in clinical and non‐clinical populations (Germer, 2009; Gilbert, 2013; MacBeth & Gumley, 2012; Neff, 2003a). The existing literature provides evidence to support the notion that compassion cultivation and practice may have a positive impact on a range of emotional, physical and life experiences whilst reducing psychopathology (Kirby, 2016). However, the majority of compassion‐based interventions are limited to predominantly Western communities, with very few being conducted with Asian populations, particularly in South Asian communities (de Zoysa et al., 2022).

### Theories of compassion

Self‐compassion is compassion given towards oneself. One of the earliest researchers to put forward a theory and measure of self‐compassion was Neff (2003a), who defined it as ‘being touched by and open to one's own suffering, not avoiding or disconnecting from it, generating the desire to alleviate one's suffering and to heal oneself with kindness’ (p. 87). Neff's interpretations, based on Buddhist teachings, emphasise that self‐compassion incorporates three components with opposing negative counterparts: mindfulness/over‐identification, common humanity/isolation and self‐kindness/self‐judgement. Neff described mindfulness as being the non‐judgemental, systematic observation of thoughts and feelings as they arise without denying or suppressing them. Over‐identification, on the other hand, is proposed as being caught up and swept away by the negative reactivity caused by distressing thoughts and feelings. Common humanity is acknowledging that pain is a shared‐human experience, whereas isolation is the perception that one is alone in their suffering. Self‐kindness is treating oneself gently and warmly, and self‐judgement is treating oneself from a cold and critical perspective when faced with failure and suffering.

In contrast, Gilbert (2013), another leading researcher in the compassion field defined compassion as ‘a sensitivity to suffering in self and others with a commitment to try to alleviate and prevent it’ (p.94). Gilbert introduced the Social Mentality Theory (SMT), which suggests that compassion emerges from the evolution of the mammalian care‐giving motivational system designed to regulate negative affect. These motivational systems are referred to as social mentalities, which evolved to overcome challenges essential for survival, such as care‐seeking and caregiving (Gilbert, 2005, 2014b). Compassion according to Gilbert (2014a) is captured by six essential competencies relating to sensitivity, sympathy, empathy, motivation, caring and distress tolerance that are experienced across three directional flows, namely self‐compassion, compassion to others and compassion from others (Gilbert, 2009).

Based on these various definitions and theoretical approaches to compassion (e.g. Gilbert, 2010; Neff, 2003a), several of clinical psychotherapeutic and general population interventions have been developed to promote compassion for the self and to/from others (Kirby, 2016). Many of these have indicated various benefits of compassion for mental well‐being, physiological health and genetic expressions (Fredrickson et al., 2008). Some of these include improved clinical outcomes (Epstein et al., 2005; Sanghavi, 2006), higher life satisfaction (Yamagata et al., 2012), quality of life (Van Dam et al., 2011), social, family and maternal support (Neff & McGehee, 2010), mindfulness (Fredrickson et al., 2008) and improved mental and personal well‐being (Feldman & Kuyken, 2011; Neff et al., 2007; Neely et al., 2009). Practising self‐compassion can reduce interpersonal problems and psychological distress (Mak et al., 2018; Schanche et al., 2011), personal pathology and psychiatric symptoms including stress (e.g. Lutz et al., 2004, 2008), depression (Shapira & Mongrain, 2010) and anxiety (e.g. Van Dam et al., 2011).

### Compassion‐based interventions

Considering the promising findings from compassion‐based intervention research on a range of presentations, an increased interest in compassion‐based interventions that specifically focus on compassion cultivation (e.g. Gilbert, 2014; Neff & Germer, 2013) has developed over the past decade. However, to date, only two meta‐analyses have investigated the efficacy of randomised controlled trials of existing compassion‐based interventions (Ferrari et al., 2019; Kirby, 2017), and only one review has provided a rigorous overview of the aims, design and evidence underpinning the existing compassion‐based interventions (Kirby, 2016). Providing evidence for the trans‐diagnostic applicability, Kirby et al.’s meta‐analysis (2017) found that compassion‐based interventions improved self‐reported compassion (*d* = .55), self‐compassion (*d* = .70) and well‐being (*d* = .51) and decreased mental health indicators such as depression (*d* = .64) and anxiety (*d* = .49). In addition, Ferrari et al. (2019) found that when compared to the control groups, self‐compassion interventions indicated significant improvements in self‐compassion (*g* = 0.75) and several other psychological outcomes including eating behaviour (*g* = 1.76), rumination (*g* = 1.37), stress (*g* = 0.67), depression (*g* = 0.66), mindfulness (*g* = 0.62), self‐criticism (*g* = 0.56) and anxiety (*g* = 0.57).

Kirby (2016) identified at least six empirically supported interventions designed with a specific focus on developing a more compassionate stance. These are Compassion Focused Therapy (CFT: Gilbert, 2014a), Mindful Self‐Compassion (MSC: Neff & Germer, 2013), Cognitively Based Compassion Training (CBCT: Pace et al., 2019), Compassion and Loving Kindness Meditations (LKM: Hofmann et al., 2011) and Compassion Meditations (CM: Wallmark et al., 2013), Cultivating Emotional Balance (CEB: Kemeny et al., 2011) and Compassion Cultivation Training (CCT: Jazaieri et al., 2013).

These interventions share certain similarities and differences, indicating the multidimensional nature of compassion (Kirby, 2016). In consideration of the similarities, all the aforementioned interventions have been influenced by the Tibetan Buddhism and incorporate some form of mindfulness practice. CFT and MSC programmes focused less on mindfulness whilst the CCT, CBCT and CEB programmes spent most of the interventions focusing on mindfulness‐based training. Importantly, all interventions include a portion of psychoeducation, providing a rationale for engaging in the compassion‐based training. All interventions also entail activities and tasks that participants are instructed to actively practice using specific compassion strategies. These practices are also similar in most interventions and contain techniques such as breathing exercises, facial and body expressions, building compassionate inner voices, compassionate letter writing and imagery tasks aimed at producing calm and soothing sensations by activating the parasympathetic system (soothing system). A homework component is also incorporated in all interventions. Interestingly, these interventions also demonstrate the ability to be delivered in group settings (Kirby, 2016).

In consideration of the differences, CFT is notably different from other compassion‐based interventions, as it is a form of psychotherapy, whereas the other interventions are simply programmes developed to increase compassion. CFT can be tailored to meet the needs of the individual, whilst the other interventions are delivered by following the prescribed session content for each session (Kirby, 2016). All interventions, except MSC, focus on compassion as a broader experience that spreads across the self and towards others, whilst MSC only focuses on self‐compassion (Neff & Germer, 2013).

It is important to note that whilst these interventions have produced encouraging results for increased well‐being (Kirby, 2016), these interventions have been developed in Western countries. For instance, the most widely used approaches such as CFT by Gilbert (2014a) and MSC by Neff and Germer (2013) originated in the UK and USA, respectively. In addition, only a limited number of RCT studies (e.g. Arimitsu, 2016; Tung, 2020) have been conducted so far, to support the use of these compassion‐based interventions (Kirby, 2016; Matos et al., 2017; Matos, Albuquerque et al., 2022; Matos, Palmeira et al., 2022). Furthermore, literature exploring compassion‐based interventions also seem to be at an infancy stage (Kirby, 2016), with the need for more rigorous trials to explore the efficacy of compassion‐based interventions across clinical and non‐clinical samples from a range of diverse backgrounds. With respect to the two existing meta‐analyses, only three of 27 studies in the meta‐analysis by Ferrari et al. (2019) and three of 17 studies by Kirby (2017) were conducted in an Asian country. Asian studies included in these reviews were also limited to China (Lo et al., 2013; Wong & Mak, 2016), Japan (Arimitsu, 2016) and Korea (Lee & Bang, 2010) suggesting that only a few countries in the East Asian cultural context have tested compassion‐based interventions. In addition, neither meta‐analysis (Ferrari et al., 2019; Kirby, 2017) assessed the potential influence of culture on the efficacy of these compassion‐based interventions.

It is important to note that whilst these interventions have produced encouraging results for increased well‐being (Kirby, 2016), these interventions have been developed in Western countries (e.g. CFT in the UK: Gilbert, 2014a, and MSC in the USA: Neff & Germer, 2013). In addition, only a limited number of RCT studies (e.g. Arimitsu, 2016; Tung, 2020) have been conducted so far, to support the use of these compassion‐based interventions (Kirby, 2016; Matos et al., 2017; Matos, Albuquerque et al., 2022; Matos, Palmeira et al., 2022). Furthermore, literature exploring compassion‐based interventions also seem to be at an infancy stage (Kirby, 2016), with the need for more rigorous trials to explore the efficacy of compassion‐based interventions across clinical and non‐clinical samples from a range of diverse backgrounds. With respect to the two existing meta‐analyses, only three of 27 studies in Ferrari et al. (2019) and three of 17 studies by Kirby (2017) were conducted in an Asian country. Asian studies included in these reviews were also limited to people in China (Lo et al., 2015; Wong & Mak, 2016), Japan (Arimitsu, 2016) and Korea (Lee & Bang, 2010) suggesting that only a few countries in the East Asian cultural context have tested compassion‐based interventions. In addition, neither meta‐analyses (Ferrari et al., 2019; Kirby, 2017) assessed the potential influence of culture on the efficacy of these compassion‐based interventions.

Therefore, the present study aimed to explore the efficacy of the existing compassion‐based interventions conducted in Asian communities. A meta‐analysis was conducted with the aim of answering the question; do compassion‐based interventions lead to increased levels of compassion in people living in Asian communities? A secondary aim was to investigate whether identified studies also reduced the additional outcomes of depression, anxiety, and stress.

## METHOD

### Protocol and registration

This meta‐analysis adhered to the general principles of the Preferred Reporting Items for Systematic Reviews and Meta‐Analyses (PRISMA: Page et al., 2021). The protocol was prospectively registered in PROSPERO, the international prospective register of systematic reviews, under the registration number CRD42020201832.

### Eligibility criteria

The primary researcher and a voluntary research assistant carried out searches independently. Studies that met the following criteria were included (a) a randomised controlled trial in which the primary focus was to purposively generate compassion or self‐compassion; (b) conducted in at least one Asian country; and (c) included at least one self‐report measure related to compassion or self‐compassion. Studies conducted across countries in the Middle East and North Africa (MENA) region were excluded as although some Middle Eastern countries are situated in the Asian continent, they are considered as countries in the MENA region (separately from other Asian countries) and share certain cultural and religious norms that are different from other Asian cultures (Alkaiyat & Weiss, 2013; Kabasakal et al., 2012). Both clinical and non‐clinical populations of all ages were included. No publication date, language or study design restrictions were applied. Eligibility criteria were based on the population, intervention, comparator, outcomes and the study type (Table 1).

**TABLE 1:** Inclusion and exclusion criteria for the review

	Inclusion criteria	Exclusion criteria
Population	Study conducted in Asian cultures	Participants in non‐Asian settings/non‐Asian
Intervention	RCTs aimed to increase compassion	Non‐RCTs/aim is not compassion (e.g. mindfulness)
Comparator	Waitlist control, active control group	No comparator/control
Outcome	Measures compassion/self‐compassion	Does not measure compassion/ self‐compassion
Studies	Published/unpublished studies, all languages	Literature reviews, opinion papers, abstracts, policy reports

### Search strategy

The systematic literature search was conducted using Scopus, Medline, Web of Science, AMED, APA PsycINFO, Ovid (EMBASE) and CINAHL databases. Cochrane Library, ProQuest for Dissertations and Theses and Open‐Dissertations databases were systematically searched to detect any relevant grey literature. The final search took place on the 10th of March 2022. The following search terms were developed with a research librarian: TI (compassion*) AND AB (random* control*) AND AB (trial OR interven* OR stud* OR program* OR therap* OR training) AND TX (Asia* OR East* OR “Eastern culture*” OR Japan* OR Chin* OR Vietnam* OR Malaysia* OR Singapore* OR “Hong Kong” OR Korea* OR India* OR Pakistan* OR Bangladesh* OR “Sri Lanka*”). Although various other interventions have integrated compassion (e.g. Mindfulness‐Based Compassion Training: Lo, 2011) or produced increased compassion (e.g. Mindfulness‐Based Cognitive Therapy: Segal et al., 2002), their primary focus is not compassion cultivation. Therefore, such interventions were excluded from the search results and only the interventions with a specific focus of compassion/self‐compassion cultivation were included.

### Data extraction

Data relating to the following study characteristics were extracted: Name of authors and year of publication, country, intervention name, design, and underpinning theory/model, aim of the study, target population, measures used, duration of the intervention, intervention tasks and the main findings of the study. For the meta‐analyses, the means, standard deviations and sample sizes for each group at pre‐ and post‐interventions were extracted.

### Analysis strategy

Version 5.4 of the RevMan software (The Cochrane Collaboration, 2020) was used for the analyses. Cohen's (1992) guidelines of small (0.2), medium (0.5) and large (0.8) effects were used when interpreting effect sizes, represented by *d*. Computations were based on a weighted‐average of the effect sizes using a random‐effects model, as it assumes that study variations can be systematic and not only due to random error (Borenstein et al., 2009). A random‐effects model is also appropriate as true effects of interventions are likely to vary depending on the sample characteristics and implementation of the intervention.

The efficacy of the interventions on compassion was compared in relation to the control groups. Control groups varied between waitlist control (WLC) and active control (AC) groups. WLC groups received no intervention, and the AC groups received a different form of intervention than the intervention groups (Kirby, 2017). In addition, a sensitivity analysis was conducted to investigate the intervention efficacy separately for the intervention groups compared with the WLC groups and intervention groups compared with the AC groups. It was expected that studies with an AC would report smaller effect sizes than studies with a WLC group, as the different interventions received by the AC groups would also influence the outcome variables (Cuijpers et al., 2016; Kirby, 2017). Another sensitivity analysis was conducted to test the intervention efficacy separately for clinical populations and non‐clinical populations when compared to the control groups. Both existing meta‐analyses (Ferrari et al., 2019; Kirby, 2017) included participants from clinical and non‐clinical backgrounds, and therefore we wanted to see whether the compassion‐based interventions would have different effects on these participant groups. Where certain studies appeared to cause large heterogeneity, further sensitivity analyses were conducted with the exclusion of those studies, to understand the heterogeneity of the studies.

In addition to testing the efficacy of the interventions on compassion, this meta‐analysis also investigated secondary outcome measures such as depression, anxiety and stress. Where one study incorporated multiple measures to measure an outcome (e.g. two measures of depression), a combined outcome was computed to produce a single mean difference by computing the average mean and standard deviation from each study (Borenstein et al., 2009). Previous meta‐analyses of compassion‐based interventions have also followed this method (Ferrari et al., 2019; Kirby, 2017). This method of providing only one effect size per study is recommended to avoid the bias of choosing one measure over another measure and to prevent attributing greater weights to studies with multiple outcomes (Borenstein et al., 2021; Ferrari et al., 2019; Kirby, 2017).

### Risk of bias within studies

Risk of bias within studies was assessed using the Cochrane risk of bias tool using Revman (Higgins et al., 2011). Critical assessments were made separately for each study for the following domains: sequence generation, allocation concealment, blinding of participants and personnel, blinding of outcome assessment, incomplete outcome data, selective outcome reporting and for other biases. Based on the information provided, each study was given a judgement of ‘high risk’, ‘low risk’ or ‘unclear’ risk of bias.

## RESULTS

### Systematic search results

**FIGURE 1:**
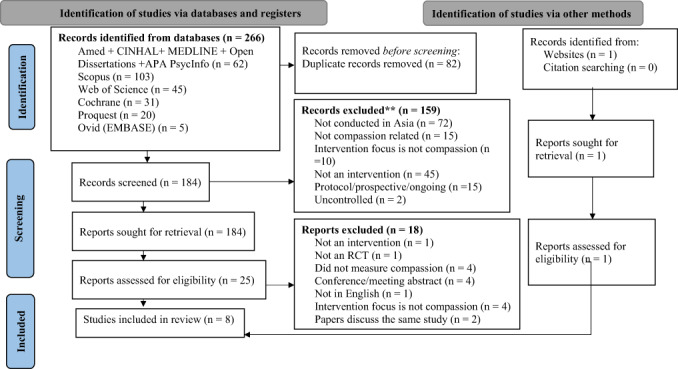
PRISMA flow diagram of study selection [Colour figure can be viewed at wileyonlinelibrary.com]

The initial database search resulted in 266 records, including 82 duplicates which were removed. Titles and abstracts of the remaining 184 papers were screened and 159 papers were excluded, as they were not related to the search. After checking the full text of 25 papers, 16 were excluded based on the eligibility criteria. Of the final nine results, three papers reported on one study (Mak et al., 2018, 2019; Yip, 2018) and therefore only one of them was retained (Mak et al., 2018). Reference lists of the chosen studies and other resources were searched for any potential studies and one further study was found on ResearchGate. This resulted in eight studies with quantitative data that were included in the meta‐analysis. All studies were allocated a number from 1 to 8 (see Table 2) and are referred to by their assigned number (e.g. ^2, 4^) going forward. Figure 1 details the search strategy.

**TABLE 2:** Intervention characteristics

No	Author, year and country	Intervention name, design, underpinning theory/model and intervention duration	Aim and target population	Measures	Comparator	Tasks	Findings
1	Anuwatgasem et al. (2020) Thailand	Mindfulness and Self‐Compassion‐based therapy (MSC) Randomised controlled trial, group, in person study design Based on MSC 7 weeks	To compare the effect of MSC on group psychotherapy on people with a DSM‐V diagnosis of Major Depressive Disorder (*n* = 23 intervention group, *n* = 11 control group)	MADRS, SCS – Thai, PSQI, HADS, Thai‐PSS‐10, RSES, WHOQOL	Pre‐test and post‐test against a standard intervention (AC group)	Activities to promote the presence of self‐kindness, common humanity and mindfulness (activities in the MSC programme—meditation, compassionate body scan etc.)	Significant decreases in the levels of depression, anxiety and stress. Significant improvements in self‐esteem and quality of life
2	Arimitsu (2016) Japan	Enhancing Self‐Compassion Programme (ESP) Randomised control, group, in person study design Based on CFT, CMT 7 weeks	To develop an ESP and test the efficacy of the programme in enhancing self‐compassion in low compassionate Japanese psychology university students (*n* = 20 intervention, *n* = 20 control group)	Acceptability questionnaire, SCS – Japanese, RSES, BDI‐2, STAI, DACS, MMS, SCES	Pre‐test post‐test and 3‐month follow‐up against a WLC group	Loving‐kindness meditation, mindfulness training, compassionate mind training using imagery, compassionate letter writing, three‐chair work, homework	Improvements in each subscale of self‐compassion except for mindfulness, and reduced negative thoughts and emotions in the ESP group
3	Guan et al., 2021 China	Self‐Compassionate Mindstate Induction (SCMI) Randomised control, online group Based on SCMI by Neff (2020) Duration not specified	To investigate online self‐compassion exercises' effectiveness in alleviating negative affect in Chinese university students during the COVID‐19 pandemic (*n* = 50 intervention, *n* = 45 control group)	Demographic information, SSCS‐L, PANAS‐negative affect, STAI‐S	Pre‐test post‐test against a neutral control group (AC group)	Writing task that contained a series of writing prompts that aimed to induce the three components of self‐compassion: mindfulness, common humanity, and self‐kindness	Significant increases in self‐compassion and decreases in negative affect when compared to participants in the control condition
4	Guo, Zhang, Mu & Ye (2020) China	Mindful Self‐Compassion Programme (MBSP) Randomised controlled trial, online study design Based on MSC 6 weeks	To explore MBSP's effects in preventing the development of PPD in women in 2nd or 3rd trimester of pregnancy with antenatal depressive or anxiety symptoms (*n* = 144 intervention, *n* = 140 control group)	MAAS, EPDS, STAI 1 and 2, BDI 2, SCS‐ Chinese, WBI of WHO‐5, The Chinese PSI, The Scales of warmth and negativity of the CPBQ, IBQ‐Short Form	Pre‐test (2nd or 3rd trimester), post‐test (3‐month post‐partum), one‐year post‐partum against a WLC group	Six sequential steps involving different types of exercises with guided instructions were performed in a stepwise way (steps/tasks not specified)	Reduced anxiety, improved mindfulness, self‐compassion, and well‐being in the MBSP group
5	Huang et al. (2021) China	Self‐Compassion Intervention Randomised controlled trial, in person, group study design Based on MBCT, CMT, and MSC 4 weeks	To test the effects of the self‐compassion intervention on future‐oriented coping and psychological distress in Chinese college students (*n* = 32 intervention group, *n* = 34 control group)	SCS, The 16‐item Future‐Oriented Coping Inventory, DASS	Pre‐test post‐test and 1‐month follow‐up against a WLC group	Psychoeducation, observing body sensations under stress, affectionate breathing meditation, loving‐kindness meditation	Increased self‐compassion and future‐oriented coping, decreased depression and stress in the intervention group
6	Mak et al. (2018) Hong Kong	Self‐Compassion Programme (SCP) 3‐arm randomised, parallel, positive‐controlled, non‐inferiority trial, online study Based MSC 4 weeks	To examine the efficacy of a mobile app‐based self‐compassion programme in improving mental well‐being and reducing distress among adults in general population (*n* = 180 intervention group, *n* = 160 cognitive behavioural group)	WHO's 50item WBI, The 6‐item K6, MAAS, SCS (13 items only), Depressed Mood and Anxiety Subscales of the ACS, 9‐item Discomfort with Ambiguity sub scale from the NCS, CSQ	Pre‐test post‐test, 3‐month follow‐up against a cognitive behavioural programme: CBP (AC group)	Compassionate body scan, affectionate breathing, loving‐kindness meditation, compassionate walking, self‐compassion break, self‐compassion journaling	Improved mental well‐being and reduced psychological distress. Enhanced mindfulness awareness at post‐programme
7	Tung (2020) Hong Kong	Mindful Self‐Compassion Programme Randomised controlled trial, in person, group study design Based on MSC 8 weeks	To increase self‐compassion and reduce stress in nursing students in Hong Kong (*n* = 33 intervention group, *n* = 44 control group)	Chinese versions of PSS, SCS, ProQOL‐5, FFMQ	Pre‐test post‐test, 1‐month follow‐up against a waitlist control group (WLC)	Meditations (affectionate breathing, compassionate body scan, loving‐kindness etc), group discussions, informal practices (e.g. soothing touch, compassionate walking, letter writing, listening etc.), homework	Reduced stress, improved self‐compassion
8	Wong and Mak (2016) Hong Kong	Self‐Compassion Writing Mixed research design with a randomised sampling method, online study Based on Neff (2009) 1 week	To examine the efficacy of self‐compassion writing on post‐writing mood, physical and psychological health in Hong Kong Chinese university students (*n* = 33 intervention group, *n* = 32 control group)	SCS (13 items only), PANAS, The 10‐item CESD, The 33‐item CHIPS, The 30‐item TMMS	Baseline, 1‐month and 3‐month follow‐up against a control writing group (AC group)	Writing on an adverse recent event and experiences about this event using mindfulness, common humanity and self‐kindness	Increased post‐writing negative affect in the self‐compassion group. Reduced physical symptoms at the follow‐ups

Abbreviations: ACS, Affective Control Scale; APS, R, Almost Perfect Scale, Revised; BDI, Beck's Depression Inventory; CAMS‐R, Cognitive and Affective Mindfulness Scale; CESD, Center for Epidemiological Studies Depression Scale; CHIPS, Cohen‐Hoberman Inventory of Physical Symptoms; CPBQ, Comprehensive Parenting Behaviour Questionnaire; CS, Compassion for others Scale; CSQ, The client satisfaction questionnaire; DACS, Depression Anxiety Cognition Scale; DASS, Depression Anxiety Stress Scale; EPDS, Edinburgh Postnatal Depression Scale; FFMQ, Five Facet Mindfulness Questionnaire; FOCS, Fear of Compassion Scale; FSCRC, Fear of Self‐Criticising/Attacking and Self‐Reassuring Scale; HADS, Hospital Anxiety Depression Scales; IBQ, Infant Behaviour Questionnaire; ISS, Internalised Shame Scale; K6, Kessler Psychological Distress Scale; LCS, Loving‐Kindness Compassion Scale; MAAS, Mindfulness Attention Awareness Scale; MADRS, Montgomery‐Åsberg Depression Rating Scale; MMS, Multiple Mood Scale; NAS, Non‐Attachment Scale; NCS, Need for Closure Scale; PANAS, Positive And Negative Affect Scale; ProQOL‐5, Professional Quality of Life Scale; PSI, Parenting Stress Index; PSQI, Pittsburgh Sleep Quality Index; PSS, Perceived Stress Scale; RRS, Ruminative Responses Scale; RSES, Rosenberg Self‐Esteem Scale; SCES, Self‐Conscious Emotions Scale; SCS, Self‐Compassion Scale; SHS, Subjective Happiness Scale; SMS, State Mindfulness Scale; SSCS, L, State Self‐compassion Scale‐Long Form; STAI, State Trait Anxiety Inventory; SWLS, Satisfaction with Life Scale; TMMS, Trait Meta‐Mood Scale; WBI, Well‐Being Inventory; WHO, World Health Organisation; WHOQOL, World Health Organisation Quality of Life.

### Quantitative results

#### Study characteristics

A total of six of the eight studies included at least one of the six compassion‐based interventions that Kirby (2016) outlined. Four studies were based on MSC (Neff & Germer, 2013) ^1, 4, 6, 7^, one was based on CFT (Gilbert, 2014a) ^2^, and another study incorporated both MSC and CMT approaches ^5^. Although not outlined in Kirby's (2016) review, the remaining two studies in the present review were based on approaches by Neff (2009), and Neff et al. (2021) with one study conducting a self‐compassion writing exercise (Neff, 2009) ^8^ and the other looking at a new approach named the Self‐Compassionate Mindstate Induction (SCMI: Neff, 2021)^3^. Four studies were delivered in person, ^1, 2, 5, 7^ and four were delivered online ^3, 4, 6, 8^, using a group^1, 2, 3, 5, 7^ or a self‐delivered approach ^4, 6, 8^.

Intervention duration varied from 1–8 weeks. One study did not specify the duration of the intervention ^3^. The authors were contacted to ascertain the information regarding the intervention duration, but no response was obtained. Four studies included a waitlist‐control group (WLC) ^2, 4, 5, 7^, and four studies included an active control group (AC) ^1, 3, 6, 8^. The AC groups received a form of intervention different to the compassion‐based interventions given to intervention groups, whilst the WLC groups received no treatment/intervention. The type of intervention received by the AC groups varied between a standard psychotherapy ^1^, a neutral writing condition ^3^, cognitive behavioural therapy ^6^ and a control writing condition ^8^. The majority of the studies also reported follow‐up data, ^2, 4, 5, 6, 7, 8^ with follow‐up periods ranging from one‐ to twelve‐month post‐intervention. Table 2 gives a summary of the study characteristics. All studies were conducted within a five‐year period (2016–2021) in several Asian countries including Thailand ^1^, Japan ^2^, China ^3, 4, 5^ including Hong Kong ^6, 7, 8^. The studies included university students ^2, 3, 5, 7, 8^, adults from the general population ^1, 6^ and pregnant women ^4^. Some studies specifically recruited adults with low self‐compassion ^2^ or symptoms of anxiety and/or depression ^1, 4^ at baseline.

### Compassion outcomes

The primary analysis was conducted to test the intervention efficacy for studies comparing the interventions with all control groups on the outcome of self‐compassion. For the sensitivity analyses, separate analyses were conducted for studies with WLC groups and studies with AC groups. The effect sizes and heterogeneity statistics for self‐compassion tested for the two categories can be found in Table 3. All studies, except one ^3^, used the SCS (Neff, 2003a, 2003b) to measure self‐compassion. Some studies used the complete scale of SCS ^1, 2, 4, 5, 7^, and other studies only used the 13 positive items of the scale ^6, 8^. One study used a recently developed State Self‐compassion Scale‐Long Form ^3^ (SSCS‐L: Neff et al., 2021).

**TABLE 3:** Post‐intervention effects on compassion and self‐compassion

Category	Outcome	*k*	*N*	*d*	*z*	*p* for *d*	*Q*	*p* for *Q*	*I* ^*2*^
Studies with a WLC	SC	4	478	0.86	4.27	<0.0001	8.95	0.03	66%
Studies with an AC	SC	4	534	0.19	2.06	0.04	3.15	0.25	5%

*Note*: d = standardised mean difference effect size; Q = test statistic for heterogeneity; k = number of samples; *N* = participants contributing to outcome; p = test for significance evaluated against .05; I^2^ = measure of degree of heterogeneity; z = z‐score.

### Compassion‐Based interventions compared with all control groups

**FIGURE 2:**
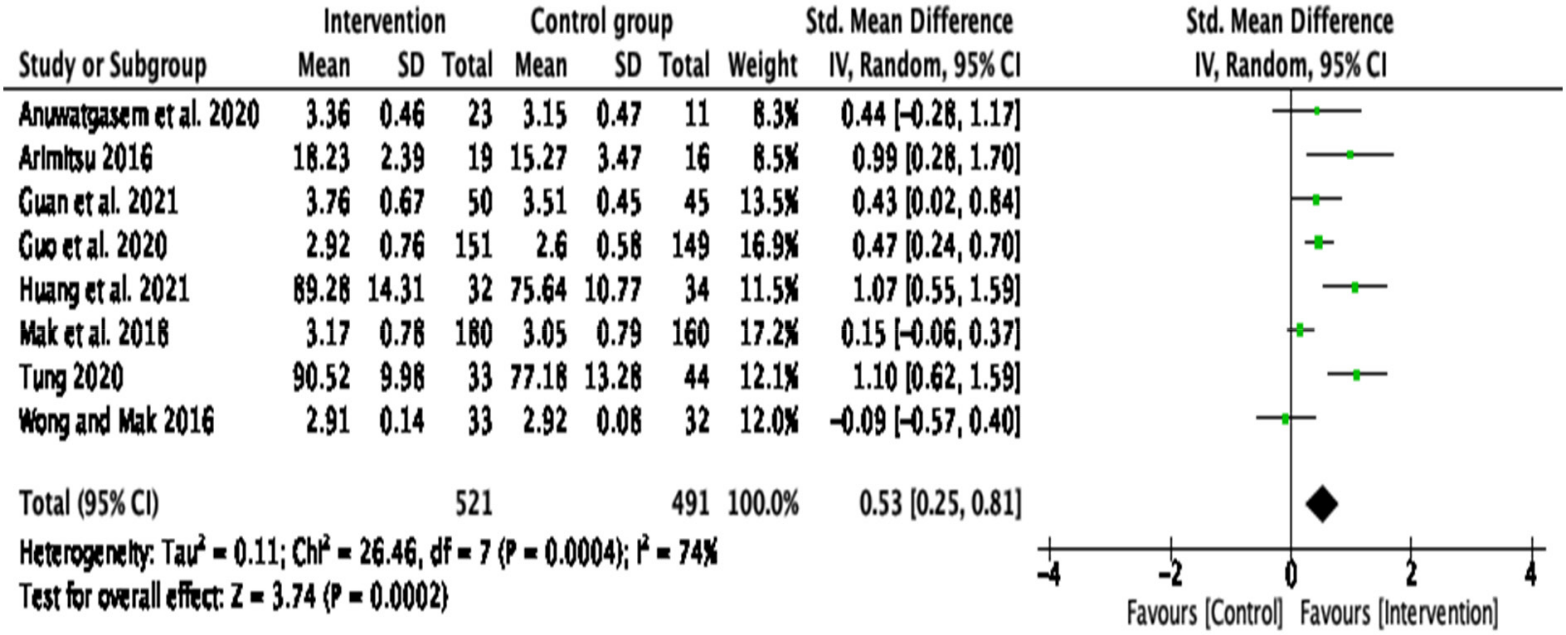
Effect of compassion‐based interventions with control groups on self‐compassion [Colour figure can be viewed at wileyonlinelibrary.com]

A significant medium effect size was reported for self‐compassion, *d* = .56, *k* = 8, 95% CI [0.25, 0.81], *p* < .001 when comparing the intervention group with a control group (including WLC and AC groups) (Figure 2). However, there was a significant amount of heterogeneity in effect sizes for self‐compassion, *Q*(7) = 24.46, *p* = .0004, *I* ^2^ = 74%.

**FIGURE 3:**
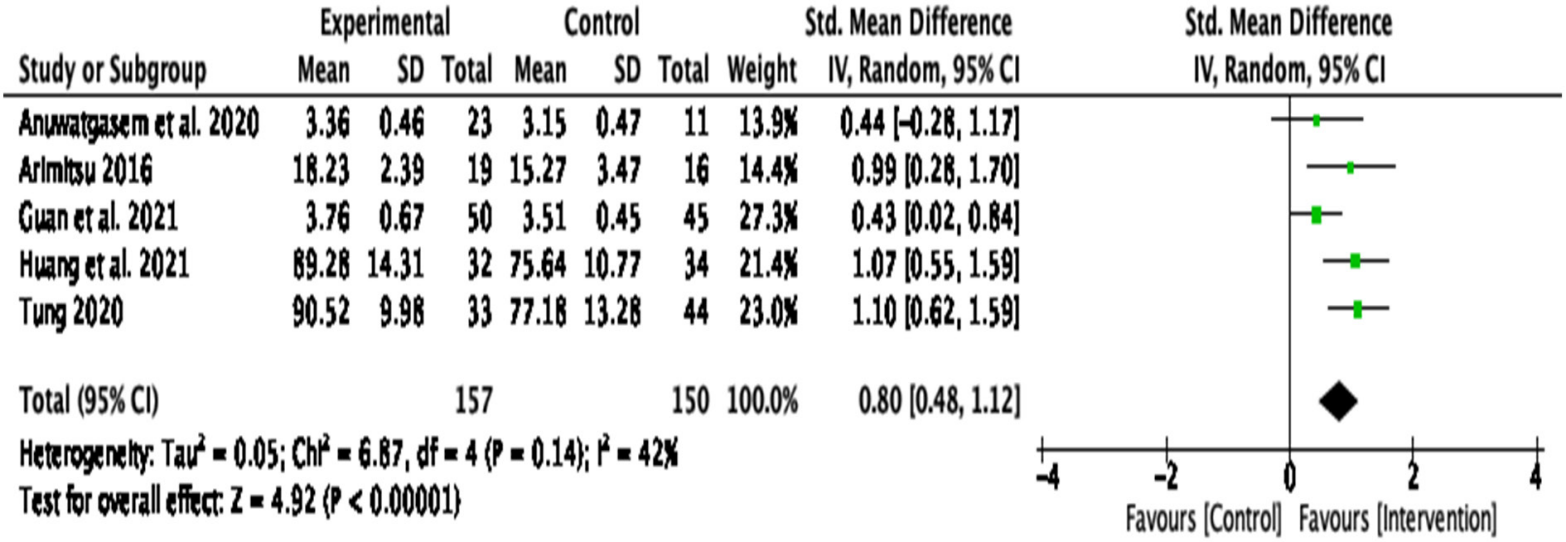
Effect of compassion‐based interventions with control groups with the omission of the three papers: Guo et al., 2020, Mak et al., 2018, Wong & Mak, 2016[Colour figure can be viewed at wileyonlinelibrary.com]

When determining what may have caused the significant statistical heterogeneity, it was clear that three papers ^4, 6, 8^(Guo et al., 2020; Mak et al., 2018; Wong & Mak, 2016) differed from the rest of the studies. Guo et al. (2020) was focused on a different target group (pregnant women in second or third trimester) and had a different intervention aim (prevention of post‐partum depression). The paper by Mak et al. (2018) differed as it was the only mobile app‐based intervention. Wong and Mak's (2016) paper also differed from the other papers as this study only used a writing exercise whereas all the other papers had multiple tasks (e.g. imagery exercises, breathing practices, etc.). To account for these differences, a sensitivity analysis was conducted with the omission of these papers (Figure 3). When these papers were removed, the results indicated a significant large effect size, *d* = .80, *k* = 5, 95% CI [0.48, 1.12], *p* < .001 with a non‐significant heterogeneity, *Q*(4) = 6.87, *p* = .14, *I* ^2^ = 42%. This implies that the remaining studies shared similar intervention characteristics.

### Compassion‐based interventions compared with waitlist control groups

**FIGURE 4:**
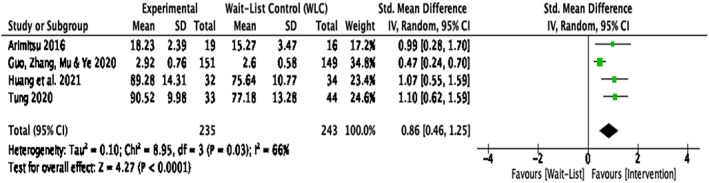
Effect of compassion‐based interventions with waitlist‐control groups on self‐compassion [Colour figure can be viewed at wileyonlinelibrary.com]

The sensitivity analysis resulted in a significant large effect size for self‐compassion, *d* = .86, *k* = 4, 95% CI [0.46, 1.25], *p* < .001 when comparing the intervention group with a WLC group ^2, 4, 5, 7^(Figure 4). There was a significant amount of heterogeneity in effect sizes for self‐compassion, *Q*(3) = 8.95, *p* = .03, *I* ^2^ = 66%.

**FIGURE 5:**
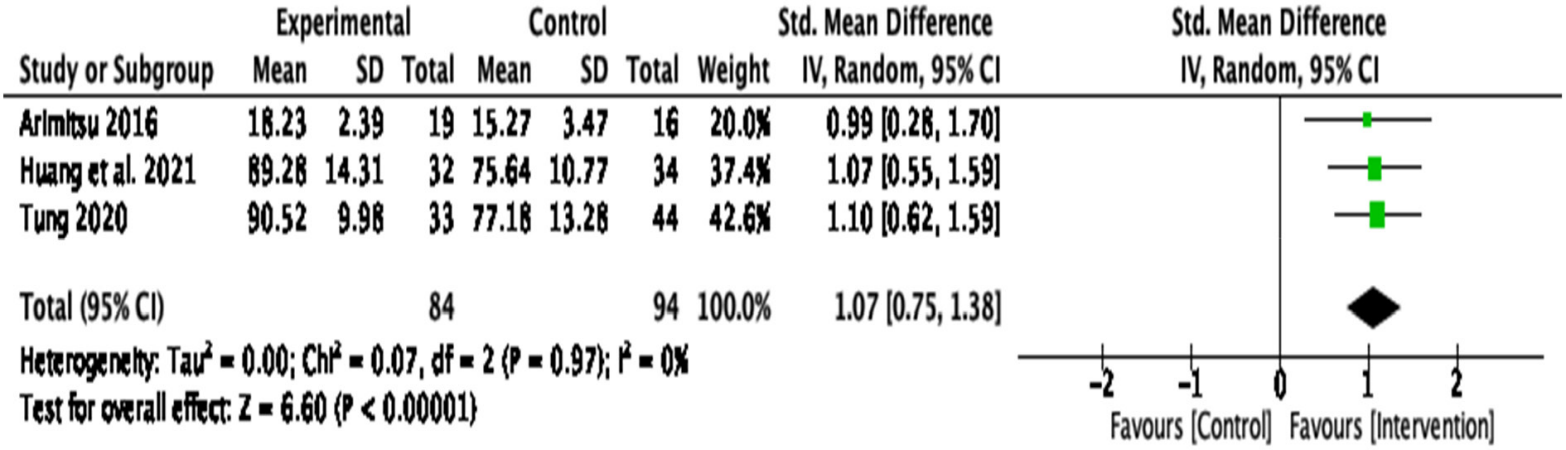
Effect of compassion‐based interventions with waitlist control groups on compassion with the omission of Guo et al. (2020) study [Colour figure can be viewed at wileyonlinelibrary.com]

The results of the analysis comparing compassion‐based interventions with WLC groups may also have been influenced by the differing target group and intervention aim of Guo et al., 2020. Therefore, to test whether this study may have affected the overall heterogeneity, a further sensitivity analysis was conducted with the omission of data of this study (Figure 5). Results indicated that there was a large effect size, *d* = 1.07, *k* = 3, 95% CI [0.75, 1.38], *p* < .001, with zero heterogeneity, *Q*(2) = 0.07, *p* = .97, *I* ^2^ = 0%.

### Compassion‐based interventions compared with active control groups

**FIGURE 6:**
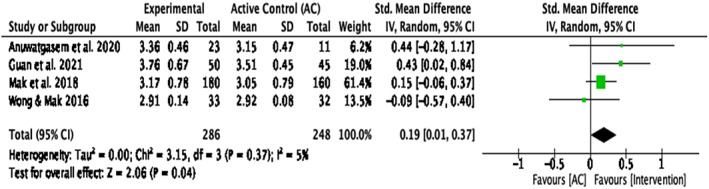
Effect of compassion‐based interventions with active control groups on self‐compassion [Colour figure can be viewed at wileyonlinelibrary.com]

When looking at intervention groups compared with AC groups ^1, 3, 6, 8^(Figure 6), a sensitivity analysis indicated a significant small effect size for self‐compassion, *d* = .19, *k* = 4, 95% CI [.01, .37], *p* = .04. Heterogeneity of variance in the effect sizes for self‐compassion was not significant, *Q*(3) = 3.15, *p* = .37, *I* ^2^ = 5%.

### Compassion‐based interventions on clinical populations

**FIGURE 7:**
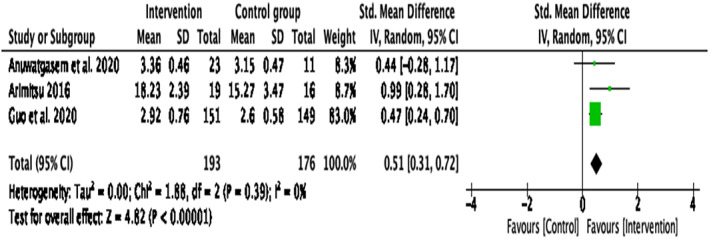
Effect of compassion‐based interventions on self‐compassion in clinical populations [Colour figure can be viewed at wileyonlinelibrary.com]

This sensitivity analysis was conducted on three papers ^1, 2, 4^ that included participants with a DSM‐V diagnosis of Major Depressive Disorder, low compassion scores (scored below the average score of 17.35 on the SCS scale) and antenatal depressive or anxiety symptoms, respectively. When looking at the effectiveness of compassion interventions on these clinical populations (Figure 7), the results indicated a medium effect for self‐compassion, *d* = .51, *k* = 3, 95% CI [.31, .72], *p* < .001. There was no heterogeneity of variance in the effect sizes for self‐compassion, *Q*(2) = 1.88, *p* = .039, *I* ^2^ = 0%.

### Compassion‐based interventions on non‐clinical populations

**FIGURE 8:**
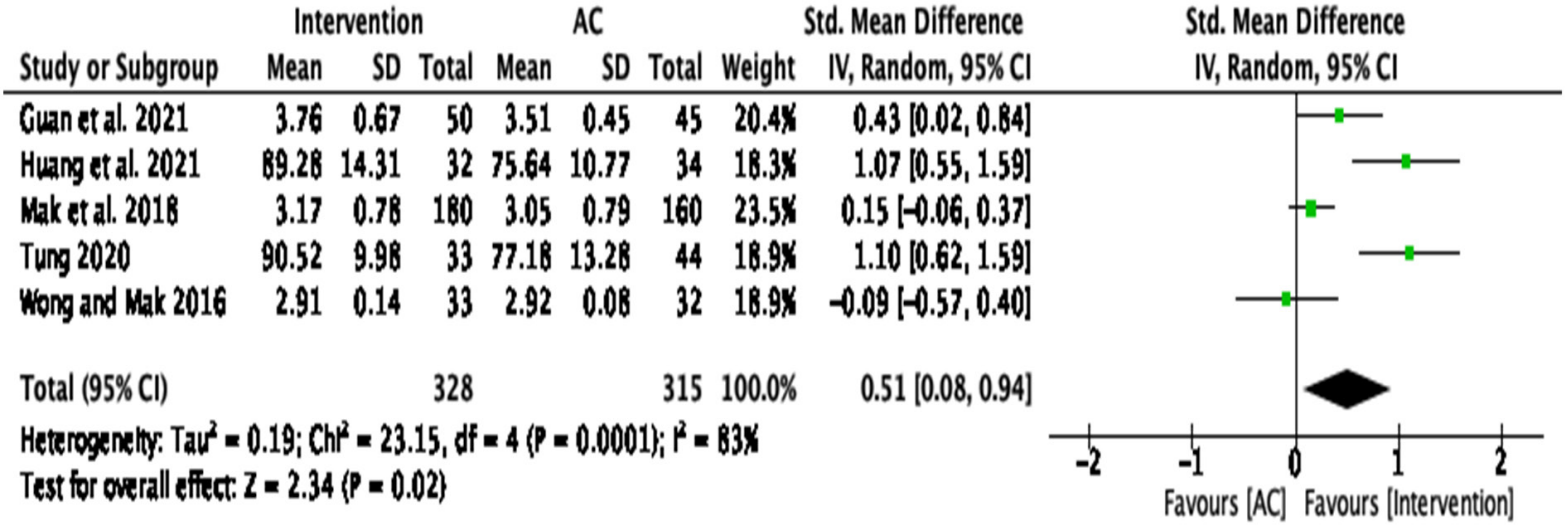
Effect of compassion‐based interventions on self‐compassion in non‐clinical populations [Colour figure can be viewed at wileyonlinelibrary.com]

**FIGURE 9:**
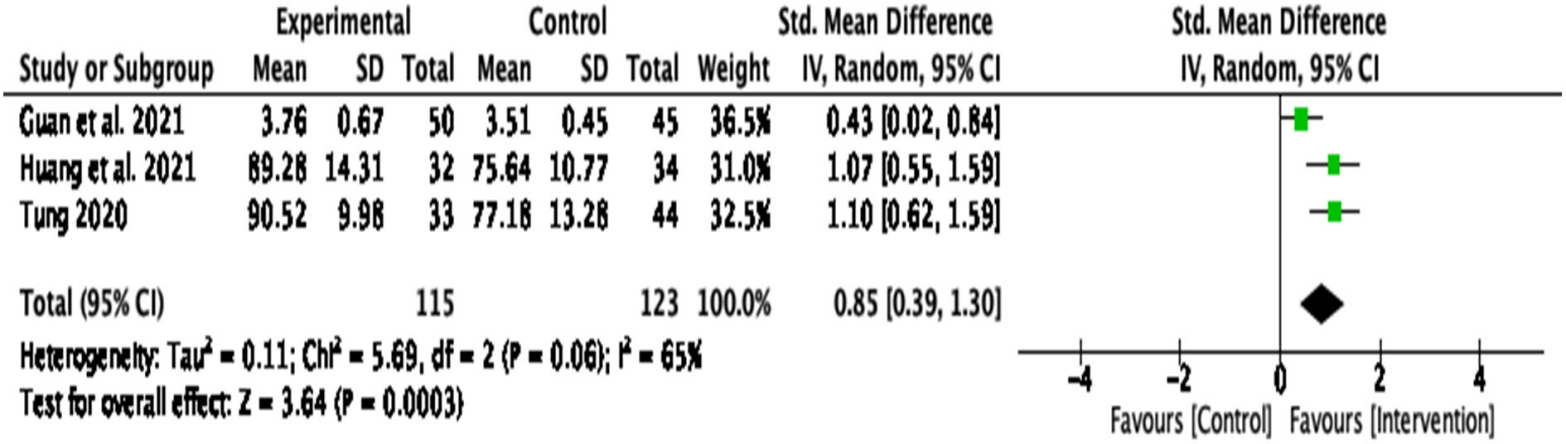
Effect of compassion‐based interventions on self‐compassion in non‐clinical populations with the omission of studies presumed to have caused heterogeneity [Colour figure can be viewed at wileyonlinelibrary.com]

This analysis was conducted on five papers ^3, 5, 6, 7, 8^, which included non‐clinical participants from universities and the general population. When looking at the effectiveness of compassion interventions in non‐clinical populations (Figure 8), results indicated a significant medium effect size for self‐compassion, *d* = .51, *k* = 5, 95% CI [.08, .94], *p* = .02. There was significant heterogeneity of variance for self‐compassion in non‐clinical populations, *Q*(4) = 23.15, *p* < .001, *I* ^2^ = 83%. Two studies were identified as causing heterogeneity ^6, 8^ (Mak et al., 2018; Wong & Mak, 2016), and, therefore, a further sensitivity analysis was conducted without these studies. This resulted in a significant large effect size, *d* = .85, *k* = 3, 95% CI [.39, 1.30], *p* < .001, with non‐significant heterogeneity, *Q*(2) = 5.69, *p* = .06, *I* ^2^ = 65% (Figure 9).

### Secondary outcomes

#### Depression

**FIGURE 10:**
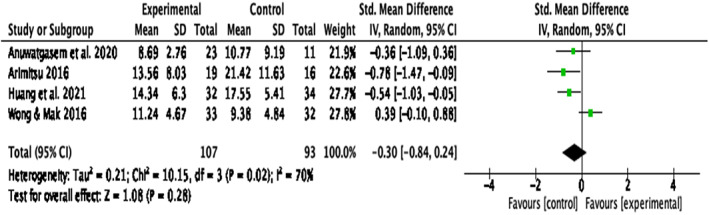
Effect of compassion‐based interventions on depression [Colour figure can be viewed at wileyonlinelibrary.com]

Four papers were included in the analysis assessing intervention efficacy on depression ^1, 2, 5, 8^. One additional paper also investigated depression ^4^ (Guo et al., 2020) but did not report these results separately so could not be included in the analysis. There was no significant intervention effect on depression, *d* = − .31, *k* = 4, 95% CI [−.84, .24], *p* = .28, but a significant large heterogeneity between the studies, *Q*(3) = 10.15, *p* = .02, *I* ^2^ = 70% (Figure 10). A sensitivity analysis omitting the studies presumed to cause heterogeneity was not conducted, as there were only four papers reporting results for depression.

#### Anxiety

**FIGURE 11:**
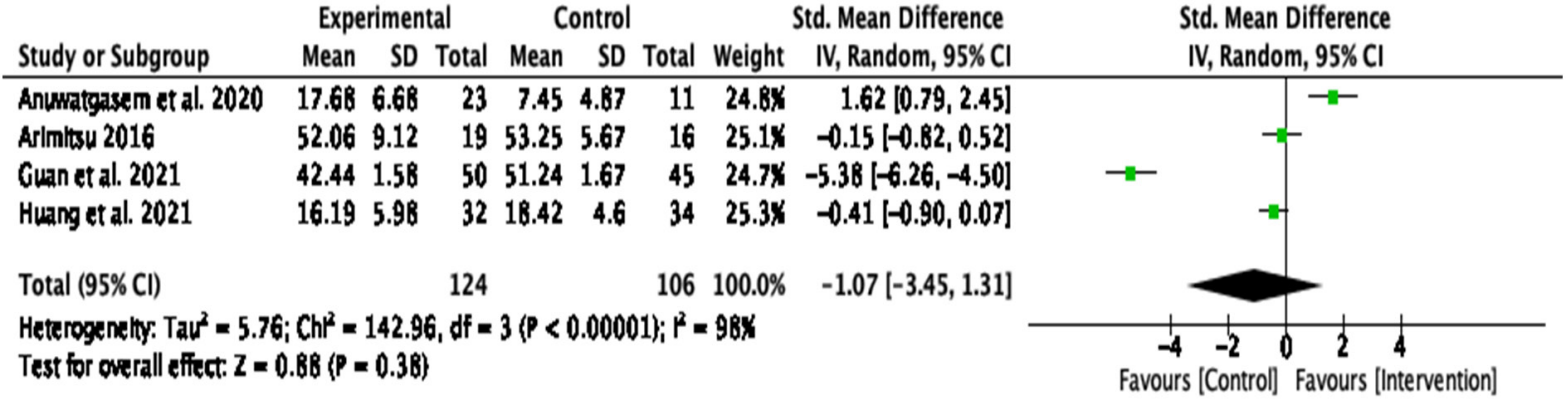
Effect of compassion‐based interventions on anxiety [Colour figure can be viewed at wileyonlinelibrary.com]

Four papers reported the intervention efficacy on anxiety ^1, 2, 3, 5^. Guo et al. (2020) ^4^ also investigated anxiety, but the study could not be included in the analysis as results were not reported separately. There was no significant intervention effect on anxiety, *d* = − 1.07, *k* = 4, 95% CI [−3.45, 1.31], *p* = .38. In addition, there was a significant large heterogeneity between the studies, *Q*(3) = 142.96, *p* < .001, *I* ^2^ = 98% (Figure 11). A sensitivity analysis omitting the studies presumed to cause heterogeneity was not conducted, as there were only four papers reporting results for anxiety.

#### Stress

**FIGURE 12:**
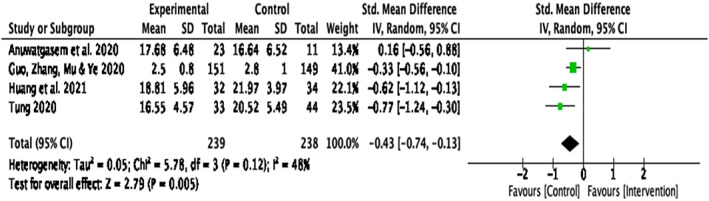
Effect of compassion‐based interventions on stress [Colour figure can be viewed at wileyonlinelibrary.com]

Four papers reported the intervention efficacy on stress ^1, 4, 5, 7^. There was a significant small intervention effect on stress, *d* = − .43, *k* = 4, 95% CI [−.75, .13], *p* = .00. In addition, heterogeneity between the studies was non‐significant, *Q*(3) = 142.96, *p* = .12, *I* ^2^ = 48% (Figure 12).

### Risk of bias within studies

**FIGURE 13:**
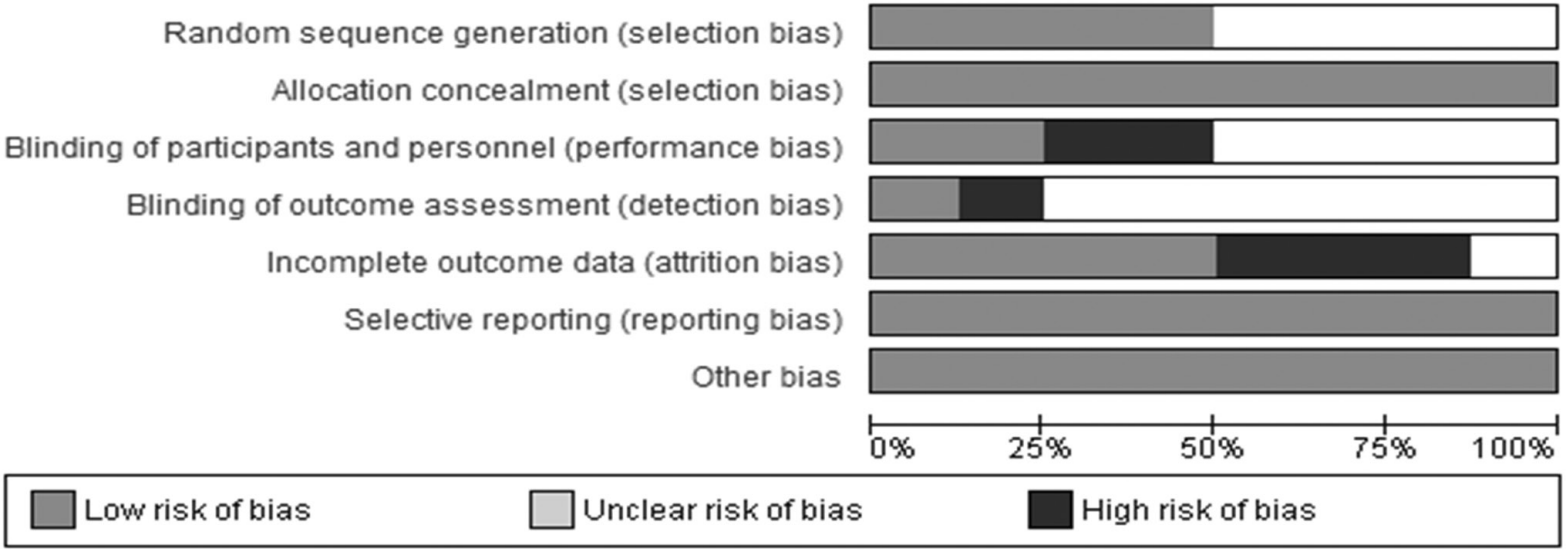
Risk of bias graph across studies

The risk of bias evaluation is displayed in Figure 13. Overall, the summary figure of risk of bias indicated a low risk of bias across studies (as indicated in the grey area). However, several studies did not report the method of randomisation ^2, 3, 5, 8^, performance bias ^5, 7^ or detection bias ^1, 2, 4, 6, 7^. Whilst all studies discussed the attrition rates and possible reasons for participant dropouts, they indicated a low risk of bias for reporting bias, selection bias, and other sources of bias.

### Risk of bias across studies

Due to the limited number of studies included in the present meta‐analysis, this study was not powered to assess publication bias across studies (Borenstein et al., 2021).

## DISCUSSION

### Compassion‐based interventions on self‐compassion

This is the first meta‐analysis to explore the efficacy and cross‐cultural applicability of compassion‐based interventions in Asian populations. This paper incorporated eight RCT studies, which gathered data from 1012 participants from Thailand, Japan and China (including Hong Kong) to answer the research question, ‘can compassion‐based interventions increase compassion in people living in Asian communities?’. The secondary aim was to investigate whether these eight studies also reduced the additional outcomes of depression, anxiety, and stress.

In relation to the first aim, results indicated that the compassion‐based interventions were effective in increasing self‐compassion among people in the Asian cultural context. However, several analyses displayed significantly large heterogeneity, reducing the confidence in the findings of the interventions included. Three studies were relatively different from the other studies across several characteristics such as study focus (Guo et al., 2020), intervention type (Mak et al., 2018) and intervention tasks (Wong & Mak, 2016). It was proposed that these studies may have contributed to the overall large variability, and when sensitivity analyses were carried out removing these studies from the analyses, the heterogeneity of studies became non‐significant, suggesting that the remaining studies had consistent findings.

Similarly, when sensitivity analyses were conducted separately for studies with WLC groups and AC groups, significant effects were reported. As predicted, effect sizes of studies including AC groups were lower when compared to studies with WLC groups (Cuijpers et al., 2016; Kirby, 2017). This implies that the AC interventions may have also increased self‐compassion in participants to some extent (Kirby, 2017). This raises the question whether the AC interventions also incorporated compassion‐enhancing tasks, or whether engaging in any intervention increases self‐compassion in general (Bishop et al., 2015).

Analyses were also conducted to investigate whether the impact of compassion‐based interventions varied between clinical and non‐clinical Asian populations. Results indicated that the compassion‐based interventions were similarly effective in both participant groups and when the three papers causing variability were omitted during sensitivity analyses (Guo et al., 2020; Mak et al., 2018; Wong & Mak, 2016), the heterogeneity of studies was non‐significant. This supports the view that compassion‐based interventions are effective when used with a wide range of participants across the Asian context (Arimitsu, 2016). However, it is important to acknowledge that these findings were based on a limited number of eight papers altogether with only three papers conducted in clinical groups. In addition, these papers were conducted across only three countries from the Southeast Asia (Thailand) and East Asia (China, Japan). Consequently, the findings of this meta‐analysis are not sufficient to draw conclusions for people in all Asian cultural contexts, and therefore future research needs be conducted in various Asian settings and participant groups (clinical, non‐clinical, student populations, public, etc.).

Kirby (2017) concluded in their meta‐analysis that there is a lack of clarity in relation to what is the most appropriate measure of self‐compassion. This lack of clarity is still evident in the current meta‐analysis, as most of the studies included used the SCS to measure self‐compassion, with some of them acknowledging the criticisms of the scale (e.g. Arimitsu, 2016; Huang et al., 2021). The Compassionate Engagement and Action Scales (CEAS: Gilbert et al., 2017) was developed to measure all three flows of compassion (self, to/from) whilst also addressing issues surrounding the SCS. Therefore, it seems fair to propose that the CEAS may be a more appropriate measure of compassion. However, none of the papers included in this meta‐analysis used the CEAS, although other recent research using this measure in Asian countries such as Japan (Asano et al., 2020) and Sri Lanka (Kariyawasam et al., 2021, 2022) have emphasised the advantages of using this measure (Asano et al., 2020). Thus, it would be useful for future research to investigate the effectiveness of compassion‐based interventions using the CEAS in Asian communities to further understand the interplay between the three flows of compassion and well‐being.

### Compassion‐based interventions on secondary outcomes

In addition to the primary outcome of self‐compassion, this study also explored whether compassion‐based interventions improved on the secondary outcome measures of depression, anxiety and stress. There was no significant impact of the compassion‐based interventions on depression and anxiety. This contradicts with previous meta‐analyses, which concluded that compassion‐based interventions were effective in reducing depression and anxiety (Kirby 2017; Ferrari et al., 2019). The present meta‐analysis indicated a large heterogeneity of study results on depression and anxiety constructs. In addition, although five studies tested intervention efficacy, one study (Guo et al., 2020) did not report on the results and therefore, could not be included in this analysis. On the other hand, there was a significant small effect of the interventions on stress whilst there was no significant heterogeneity in the study results. Although the individual studies reporting these results indicated that the interventions did in fact improve depression, anxiety and stress, only a few studies of the eight papers investigated these constructs and the large variability in the previously mentioned studies may have resulted in the non‐significant impact on depression and anxiety, and the small significant effect on stress. Therefore, it is difficult to determine the impact of compassion‐based interventions on these outcomes and further studies need to be explored to draw conclusions.

### Strengths and limitations

A strength of this meta‐analysis is that it only included papers with a specific focus on compassion cultivation and excluded interventions that prioritised other elements such as mindfulness (e.g. Mindfulness Based Stress Reduction, Mindfulness Based Cognitive Therapy). One paper used in this meta‐analysis, however (Huang et al., 2021), based their intervention on both MSC (developed by Neff & Germer, 2013) and CMT (developed by Gilbert, 2009) approaches. It was therefore not possible to differentiate whether self‐compassion increased due to the mindfulness element, the compassion element or both, when assessing the efficacy of compassion interventions based on this integrated approach (Kirby 2017).

Overall, this review highlights that the lack of research exploring the effectiveness of compassion‐based interventions in Eastern cultures, as there were only three Asian countries that have researched this area so far. This was surprising given that many Asian countries such as Sri Lanka are familiar with compassion practice due to the significant Buddhist influence in such cultures (Kariyawasam et al., 2021, 2022).

Whilst the rigorous inclusion criteria helped to choose studies with higher methodological quality, this was also a limitation of this meta‐analysis. This was because it lead to the exclusion of several studies which were either not RCTs despite being compassion‐based interventions to promote compassion (e.g. Finlay‐Jones et al., 2018; Noh & Cho, 2020; Yeung et al., 2022) or did not include compassion measures to assess the intervention efficacy (e.g. Lo et al., 2015).

Another limitation this meta‐analysis discovered was that despite a wide search strategy, all the included studies only assessed self‐compassion, disregarding the other two flows, namely compassion to others, and compassion from others (Gilbert et al., 2017). Ferrari et al. (2019) also narrowed their search to self‐compassion‐based interventions only, when conducting their meta‐analysis of compassion‐based interventions. Gilbert et al. (2017) argued that compassion is not only felt for the self but also towards and from others. Studies have discussed how these flows interact with one another (Rashid et al., 2021) and are also linked with increased well‐being (Asano et al., 2020; Gilbert et al., 2017).

In addition, findings of the studies included in this review were mostly limited to non‐clinical populations (e.g. Arimitsu, 2016; Guan et al., 2021), indicating the need for further research among both clinical and non‐clinical populations in more Asian countries. The majority of the studies (five papers) collected data from university students, which also brings into question the generalisability of the findings to the larger context. Despite the common use of small underpowered sample sizes in many evidence‐based interventions (Kirby 2017), this review noted that the papers generally included a small sample size, which also limits the generalisability of the findings. The limited number of RCTs meant that a funnel plot was not suitable to assess the risk of publication bias (Higgins et al., 2011).

Similar to Kirby (2017), this meta‐analysis did not find studies that assessed compassion using heart rate variability and other bodily measures that would have increased the understanding of intervention efficacy (Luo et al., 2018; Tian et al., 2020) at a physiological level. Furthermore, the RCTs included a range of self‐reported levels of depression, anxiety and stress measures, which may be particularly problematic due to the stigma of mental illness in Asian cultures (Wong & Mak, 2016). It seems fair to propose that future research should focus on using physiological measures (Finlay‐Jones et al., 2018) in addition to self‐report measures to help build a comprehensive understanding of the efficacy of compassion‐based approaches.

The findings of this meta‐analysis should be interpreted with caution when considering the impact of compassion‐based interventions in Asian communities, as this meta‐analysis was limited to only eight studies which were gathered from participants across three Asian countries. Therefore, whilst this was the first attempt to conduct a meta‐analysis for people in Asian communities, this study cannot be generalised to all people in the Asian cultural context.

Additionally, this meta‐analysis comprised studies that varied in multiple components such as the intervention duration, content and targeted population. Thus, prospective interventions could investigate the content and structure of compassion‐based interventions, to determine the most suitable intervention for their targeted populations.

### Clinical implications

This review found potentially positive effects on self‐compassion when using compassion‐based interventions in Asian populations. Both online (e.g. Mak et al., 2018; Wong & Mak, 2016) and in‐person approaches (e.g. Huang et al., 2021; Tung, 2020) were found to be effective in increasing self‐compassion and well‐being and in reducing negative affect in Asian communities. Given that many people from Asian backgrounds do not seek help for their emotional well‐being due to high shame and criticism, stigma and other help‐seeking barriers in their societies (Kariyawasam et al., 2022; Mak et al., 2019), it seems fair to propose that online interventions such as the Self‐Compassion App (Beaumont & Irons, 2021) maybe more appropriate for people in Asian communities. This would also reduce the need and cost of training clinicians or other specialist staff to deliver compassion‐based approaches in cultures where there are high levels of poverty and limited funding for mental health clinics. Online approaches are found to be more interactive, cost‐effective, quick, scalable and convenient (Chi, 2013; Mak et al., 2018). In addition, with the rapid increase of mental health complications due to the recent COVID‐19 pandemic (Pfefferbaum & North, 2020), and with the new working from home environment, online interventions for facilitating well‐being would be particularly convenient, timely and effective. Thus, the applicability and efficacy of online compassion‐based compassion approaches are avenues of future research.

## CONCLUSION

This meta‐analysis explored the efficacy of compassion‐based interventions in Asian populations to increase levels of compassion. The results suggested that compassion interventions increased self‐compassion in clinical and non‐clinical samples, providing evidence for the trans‐diagnostic (Anuwatgasem et al., 2020) and cross‐cultural application of these approaches (Tung, 2020). Self‐compassion increased with significant effect sizes, in studies with WLC groups when compared to studies with AC groups, indicating that active‐control conditions may have also increased self‐compassion. Prospective studies are encouraged to develop interventions, carefully selecting appropriate measures and assessing physiological changes to obtain outcomes that are more informed. Although there were several limitations including the limited number of studies and sample sizes, this meta‐analysis encourages the use of compassion‐based interventions to promote compassion and well‐being in Asian communities.

## AUTHOR CONTRIBUTIONS

**Lasara Kariyawasam:** Conceptualization; data curation; formal analysis; investigation; methodology; project administration; resources; software; validation; visualization; writing – original draft. **Margarita Ononaiye:** Conceptualization; investigation; methodology; project administration; resources; supervision; validation; visualization; writing – review and editing. **Chris Irons:** Conceptualization; methodology; project administration; resources; supervision; validation; visualization; writing – review and editing. **Sarah E. Kirby:** Conceptualization; formal analysis; investigation; methodology; project administration; resources; software; supervision; validation; visualization; writing – review and editing.

## CONFLICT OF INTEREST

The authors declare that there are no conflicts of interest.

## Data Availability

Data sharing is not applicable to this article as no data sets were generated or analysed during the current study.
